# Depressive symptoms homophily among community-dwelling older adults in japan: A social networks analysis

**DOI:** 10.3389/fpubh.2022.965026

**Published:** 2022-09-20

**Authors:** Ayako Morita, Yoshimitsu Takahashi, Kunihiko Takahashi, Takeo Fujiwara

**Affiliations:** ^1^Department of Global Health Promotion, Tokyo Medical and Dental University, Tokyo, Japan; ^2^Department of Health Informatics, Kyoto University School of Public Health, Kyoto, Japan; ^3^Department of Biostatistics, Tokyo Medical and Dental University, Tokyo, Japan

**Keywords:** network analysis, birds of a feather, apathy, suicidal ideation, East Asia

## Abstract

Late-life depression is one of the most common mental illnesses that cause serious consequences, but the majority do not reach out for mental health services and relapses are common. The present study investigated profiled similarity of older adults' social networks in terms of depressive symptoms. In 2017, we distributed questionnaires inquiring about confidants in the community, depressive symptoms based on the 15-item Geriatric Depression Scale (GDS-15), and demographic and functional characteristics to all the community-dwelling older adults under the national insurance system in Wakuya City (Miyagi prefecture, Japan). Applying the Exponential Random Graph Model, we estimated the likelihood of a confidant relational tie by the similarity of overall and specific depressive symptoms within 217,470 potential ties among 660 respondents eligible for analysis. The overall depressive symptom homophily was marginally significant (*p* < 0.10), indicating that the likelihood of a confidant relational tie between two community-dwelling older adults was decreased by 5%, with one point increase in their difference in the total number of depressive symptoms (odds ratio [OR], 0.95; 95% confidence interval [CI], 0.90–1). Focusing on specific domains of depressive symptoms, we found significant apathy homophily (*p* < 0.05) but no significant suicidal ideation of homophily. The results indicated that there is a 19% decrease in the likelihood of a confidant relational tie between two community-dwelling older adults by one point increase in their difference in the total number of apathy symptoms (OR, 0.81; 95%CI, 0.67–0.98) but no change by increasing the difference in their total number of suicidal ideation symptoms (OR, 1; 95%CI, 0.87–1.14). These findings suggest depressive symptom homophily, particularly with respect to apathy domains, in confidant social networks of community-dwelling older adults, and the importance of network intervention in preventing late-life depression.

## Introduction

Depression is a common mental disorder in older adults with a prevalence ranging from 4.5% to 49% (1). Older adults with depression have been found to suffer from poor general health, lower health-related quality of life (2), and increased risk of developing physical and cognitive frailty, dementia, and death (3, 4). Late-life depression is imposing enormous costs on individuals, their families, and aging societies (5). It is urgent to understand why the majority of older adults with depression are not reaching out for mental health care and are left untreated (6), and relapses and chronicity are common (7).

As the Greek philosopher, Aristotle, said, “man is, by nature, a social animal,” we rely on networks of relationships with family, friends, and other individuals in society to survive and thrive. Social network changes across the life span, and the older adults' social networks have been described as relatively smaller in size but closer in social proximity, mainly due to shrinking peripheral networks (8, 9). Social network studies have reported that similarity serves as a strong foundation for the formation and maintenance of strong ties, such as best friends and marital couples (10–12), and negative emotions could spread *via* those social ties within a community (13, 14). As documented in case reports of suicide among depressed older adult couples [M. D. (15–18)], shared characteristics and co-occurrence of depression in social networks could hinder appropriate treatment to overcome depression.

To date, however, there is a limited number of empirical studies that investigated older adults' social networks between individuals from not only one side but from both sides (19). Older adults' social networks have been found to share some health-related characteristics, such as smoking behavior, physical inactivity (20), and logical memory function (21), but it is unknown whether older adults' social networks also share depressive emotional attributes.

Understanding the shared emotional attributes of older adults' social networks would help design interventions to prevent late-life depression. The present study aimed to investigate depression homophily by analyzing whether similarity in depressive symptoms is associated with a confidant relational tie in dynamic data collected among older adults living in one community.

## Materials and methods

### Participants

Between August and September 2017, a self-reported survey was mailed to 4,902 older adult beneficiaries of the national health insurance or late-stage medical care system in Wakuya City, Miyagi Prefecture, Japan, which covered about 90% of the residents aged 65 and over (22). The present study analyzed the responses of 660 community-dwelling older adults who provided information on their depressive symptoms and confidants (i.e., intimate person to whom one feels close and whom one trusts).

The research Ethics Committee at Tokyo Medical Dental University approved the study protocols and the participants provided written informed consent. All methods were carried out in accordance with the Declaration of Helsinki and Japan's ethical guidelines for epidemiologic research (23).

### Outcome: Confidant social networks

Confidant relations can be directed (i.e., not necessarily reciprocated). We defined confidant social networks as relationships, in which information and advice are likely shared in response to self-disclosure about personal problems from one side or both sides. As the first step, we asked the participants to list all the household members and their relationships, marital satisfaction, and presence of confidants. We identified all co-residing older adults as confidants unless the participants reported poor marital satisfaction (i.e., more than six points on a 10-point Likert scale) and absence of confidants. In such cases, we did not identify opposite-sex individuals with <15 years of age difference as confidants. In the second step, we asked the participants to list all the non-coresiding persons in the community to whom they disclose their worries, and identified the persons who met the following criteria as confidants: (1) first and family names were spelled the same in either Japanese kanji or hiragana; (2) same-sex; (3) no more than 3 years of age difference; (4) same residential district but not the same residential address; and (5) no other beneficiaries of the national health insurance and late-stage medical care system that met the first four criteria.

### Depressive symptoms

A 15-item version of the Geriatric Depression Scale (GDS-15) in Japanese (24) was administered. GDS-15 assesses the presence of multi-dimensional symptoms of depression, such as dysphoric mood, withdrawal-apathy-vigor, hopelessness, subjective memory complaints, and anxiety, with dichotomized questions, and a higher total score (0 to 15) indicates more depressive symptoms (25).

To date, two sub-scales had been proposed and validated in measuring distinctive dimensions of depression for clinical use. One of them is the three-item apathy subscale (26), which measures the apathy dimension of depression based on three items from GDS-15 (i.e., “Have you dropped many of your activities and interests?,” “Do you prefer to stay at home rather than go out and do new things?,” and “Do you feel full of energy?”). The other is the five-item suicidal ideation subscale that measures the suicide ideation dimension of depression based on five items from GDS-15 (“Do you feel that your life is empty?,” “Do you feel happy most of the time?,” “Do you think it is wonderful to be alive now?,” “Do you feel pretty worthless the way you are now?,” and “Do you feel that your situation is hopeless?”) (27). The apathy-subscale scores ranged from 0 to 3 and the suicidal ideation subscale score ranged from 0 to 5.

The similarity in depression symptoms between participants was measured by computing their absolute difference in the GDS-15 total scores, the apathy subscale scores, and the suicidal ideation subscale scores.

### Covariates

We measured demographic and health characteristics that are closely associated with social isolation and late-life depression. In addition to age and sex, we measured the number of household members (0 = one person or living alone, 1 = more than 2 persons), educational attainment (0 = less than 6 years, 1 = 6-9 years, 2 = 10-12 years, and 3 = more than 13 years), and presence of subjective memory complaint (0 = absent, 1 = present) and disability (0 = absent, 1 = present). Subjective memory complaint (SMC) was measured by the SMC scale (28), which asked the participants to report the frequency of experiencing forgetfulness about a person's name, place, plan, and today's date or presence of other severe memory problems, such as forgetting to turn off the stove. The score range was between 4 and 28, and a higher score indicated a higher degree of SMC. The presence of disability was measured by the 13-item Tokyo Metropolitan Institute of Gerontology Index of Competence (TMIG-IC), and we considered less than a full score (<13) as an indication of disability (29).

### Statistical analysis

Our data was directed network data, and we regarded there was a confidant tie between two participants when at least one of them was a confidant to the other, and calculated *density* with the following formula: [observed number of ties excluding the duplicates due to reciprocated relations] ÷ [maximum number of ties that potentially arise among the participants, i.e., *n* (*n*−1)/2].

To estimate a probability of a confidant relational tie, we built three multiple logistic regression models. The outcome was the presence (1) or the absence (0) of a confidant relationship between nodes (i.e., *Y*_*ij*_ = 1 when actor *i* regarded actor *j* as a confidant, and *Y*_*ij*_ = 0 when actor *i* did not regard actor *j* as a confidant). Exposure was similar on the total and subscale GDS-15 scores between actors *i* and *j*. We further added several individual attributes (i.e., sex, age, lone-household status, educational attainment, subjective memory complaints, higher-level functional independence, and depressive symptoms) and network structure (i.e., density, reciprocity) in the model that may affect the likelihood of a confidant tie and difference from the other participants in depressive symptoms. We performed Exponential-family Random Graph Models (ERGM) using Markov Chain Monte Carlo (MCMC simulation), the modified logistic regression model, allowing the estimation of the likelihood of a tie while adjusting for network structural effects (21, 30–32). Using exponentiating (exp) coefficient and standard error, we computed the log-odds likelihood of a confidant relational tie for the exposure, and the 95% confidence interval (CI) was computed by exp (coefficient ± two-sided 95% confidence limit for a z-score^*^standard error) (21).

We used the ergm package (33) and the igraph package (34) on R version 4.0.3 to perform the analyses above, and chose the Fruchterman-Reingold's force-directed placement algorithm to visualize the networks.

## Results

In the survey, 385 (58.3%) participants reported they had at least one confidant of any age in the community. Table 1 presents a summary of the demographic and health composition, and negative emotion levels of the participants. There were slightly more women (*n* = 351, 53.3%), and the mean age was 76.3 years old. The majority (*n* = 563, 85.1%) was living with a family. Approximately half of the participants completed 10–12 years of formal education. The mean score of subjective memory complaints was 10.5, and 45.5% were free of disability. The mean score of GDS-15 for overall depressive symptoms, apathy, and suicidal ideation were 5, 1.4, and 1.3, respectively.

**Table 1:** Characteristics of participants (*n* = 660).

	***n***	**%**	**Mean (SD)**
Age	660		76.2 (7.3)
Sex (female)	351	53.2	
Living arrangement (with a family)	563	85.1	
Educational attainment			
Equivalent or <9 years	215	32.6	
10–12 years	309	46.8	
13 years+	127	19.2	
Other (including unknown)	9	1.4	
Subjective memory complaints^b^	660		10.5 (4.9)
Disability			
No	100	45.2	
Yes	316	47.9	
Missing	46	7.0	
Geriatric depression status (GDS-15 score range)			
Not depressed (0–4)	350	53.0	
Mildly depressed (5–9)	211	32.0	
Severely depressed (10–15)	99	15.0	
Depressivity (GDS-15 total score)	660		5.0 (3.8)
Apathy (GDS-15 subscale score)	660		1.4 (1.1)
Suicidal ideation (GDS-15 subscale score)	660		1.3 (1.5)

16 missing responses were imputed with the mean score; Correlation between apathy and suicidal ideation was 0.55 (95% confidential interval: 0.49–0.60).

Network-level descriptive statistics are presented in Table 2. There were 300 directed ties among the participants. Of those, 155 (51.7%) were established with older adults outside of the household and 128 (42.7%) were established with older adults within the household. More than half of the ties (n = 180, 61.5%) were reciprocated. A density was 0.001 (= one confidant relational tie per 1,000 older adults in the same community).

**Table 2:** Characteristics of confidant network (directed ties) among participants.

	***n* or %**
Density (%)	0.001
Number of vertices with more than one tie	297
Total confidant ties	300
Inside the household	134
Outside the household	166
**Edge type**	
Unique	120
Reciprocal (two-way) ties	180
**Group characteristics**	
Number of social networks (range of the size)	96 (2–36)
Mean geodesic distance within the participants	2.8

**Figure 1:**
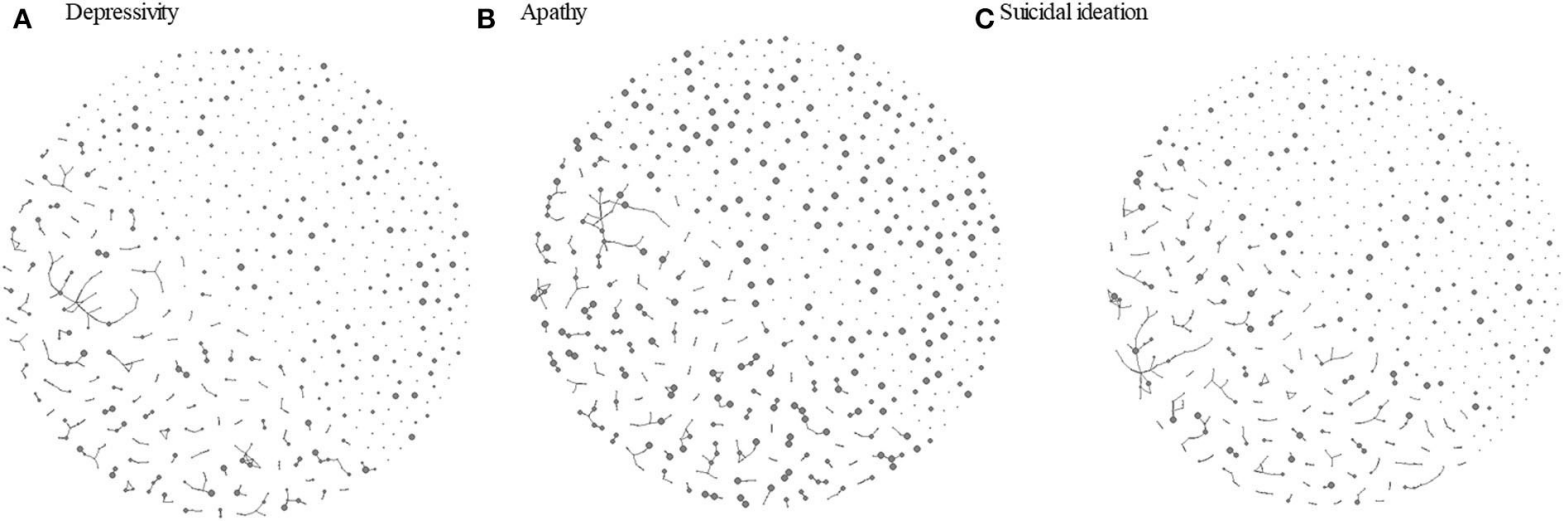
Visualization of confidant network among older adults in a community by the level of depressive status. **(A)** Apathy. **(B)** Sucide ideation. **(C)** Circle size represents the total score on GDS-15 (divided by 3 to improve visualization), a subscale score on apathy and a subscale score on sucide ideation with a bigger size represents a higher level of respective negativ emotion characteristics. Arrow heads indicate who is directing the tie toward whom.

We present a visualization of negative emotions in the confidant network in Figure 1. Each node represents a participant with an arrow indicating a relationship to be one-way or reciprocated, and the node size corresponded to scores on depressive symptoms (Figure 1A), suicidal ideation (Figure 1B), and apathy score (Figure 1C). While the score difference ranged from 0 to 14 points for depressive symptoms, 211 (68%) ties fell within five points difference and 28 (9%) ties displayed the same score. For suicide ideation and apathy subscales, the score difference ranged from 0 to 5 and 0 to 3, respectively, and there were 96 (31%) exactly matched cases for suicidal ideation and 112 (36.1%) for apathy.

Table 3 presents the estimation of the effects of overall depressive symptom homophily on the probability of a confidant relational tie in a full model of the ERGM analyses. A confidant relational tie was marginally associated with similarity on depressive symptoms (coefficient, −0.052; *p* = 0.07) and significantly associated with presence of disability (coefficient, 0.38; *p* < 0.001) and reciprocity (coefficient, 8.02; *p* < 0.001). The result indicates that the likelihood of a confidant relational tie between two community-dwelling older adults decreases by 5% with one point increase in their difference in the total number of depressive symptoms (odds ratio [OR], 0.95; 95% confidence interval [CI], 0.90–1) adjusted for the individual attribute and network structural effects.

**Table 3:** Multivariate logistic regression on the likelihood of a confidant relational tie by similarity in overall depressivity, individual attributes, and network variables among the community-dwelling older adults.

	**Coefficient**	**SE**	***p*-value**	**OR (95%CI)**
**Homophily effects**			
Similarity in overall depressivity	−0.052	0.029	0.07	0.95 (0.90, 1.00)
**Individual attributable effects**		
Sex (ref: male)	0.01	0.12	0.93	1.01 (0.80, 1.28)
Age (ref: <75 years-old)	0.13	0.13	0.31	1.14 (0.88, 1.47)
Living arrangement (ref: living alone)	−0.19	0.18	0.29	0.83 (0.58, 1.18)
Disability (ref: no)	0.38	0.14	<0.001	1.46 (1.11, 1.92)
Educational attainment (ref: equivalent or <9 years)
10–12 years	0.07	0.12	0.55	1.07 (0.85, 1.36)
13 years+	−0.001	0.14	0.99	1.00 (0.76, 1.31)
Depressive symptoms	0.0066	0.018	0.72	1.01 (0.97, 1.04)
**Social structural effects**		
Edges	−8.67	0.37	<0.001	
Reciprocity	8.02	0.46	<0.001	

Table 4 presents the estimation of the effects of specific domains of depressive symptoms. A confidant relational tie was significantly associated with similarity on apathy (coefficient, 0.21; *p* = 0.038), age (coefficient, −1.45; *p* < 0.001), living with family (coefficient, 0.65; *p*<*0.001*), presence of disability (coefficient, 0.38; *p*<*0.001*), level of apathy (coefficient, 0.22; *p*<*0*.01), and reciprocity (coefficient, 25.4; *p*<*0.001*) and marginally associated with education (coefficient, 0.35; *p* = 0.084) on a confidant relational tie. The result indicates that the likelihood of a confidant relational tie increases by 23% with one point decrease in their difference in the total number of apathy symptoms (OR, 0.81; 95%CI, 0.67 – 0.98). On the other hand, a confidant relational tie was not significantly associated with suicidal ideation. In the model, which included suicide ideation variables, a confidant relational tie was significantly associated with disability (coefficient, −0.35; *p* < 0.001) and reciprocity (coefficient, −9.20; *p*<*0.001*) but it was not significantly associated with similarity in suicide ideation (coefficient, 0.0043; *p* = 0.95). The OR of a confidant relational tie with one point decrease in their difference in the total number of suicide ideation symptoms was 1 (95%CI, 0.87–1.14).

**Table 4:** Multivariate logistic regression on the likelihood of a confidant relational tie by similarity in specific domains of depressive symptoms, individual attributes, and network variables among the community-dwelling older adults.

	**Model 1 (Apathy homophily test)**				**Model 2 (Suicidal ideation homophily test)**			
	**Coefficient**	**SE**	***p*-value**	**OR (95%CI)**	**Coefficient**	**SE**	***p*-value**	**OR (95%CI)**
**Homophily effects**								
Similarity in the level of apathy	−0.21	0.099	0.038	0.81 (0.67, 0.98)		
Similarity in the level of suicidal ideation					−0.0043	0.07	0.95	1.00 (0.87, 1.14)
**Individual attributable effects**								
Sex (ref: male)	0.07	0.13	0.58	1.07 (0.83, 1.40)	0.01	0.11	0.92	1.01 (0.81, 1.25)
Age (ref: <75 years-old)	−1.45	0.15	<0.001	0.23 (0.17, 0.31)	0.17	0.11	0.12	1.19 (0.96, 1.47)
Living arrangement (ref: living alone)	0.65	0.18	<0.001	1.92 (1.30, 2.73)	−0.09	0.13	0.47	0.91 (0.71, 1.18)
Disability (ref: no)	0.38	0.17	<0.001	1.46 (1.00, 2.04)	0.35	0.12	<0.001	1.42 (1.10, 1.80)
Educational attainment (ref: equivalent or <9 years)								
10–12 years	0.13	0.16	0.43	1.14 (0.83, 1.56)	0.17	0.13	0.19	1.19 (0.92, 1.53)
13 years+	0.35	0.2	0.084	1.42 (0.96, 2.10)	0.25	0.16	0.11	1.28 (0.94, 1.76)
Level of apathy	0.22	0.074	0.003	1.24 (1.07, 1.44)		
Level of suicidal ideation					0.058	0.04	0.11	1.06 (0.98, 1.15)
**Social structural effects**								
Edges	−8.5	0.48	<0.001		−9.2	0.27	<0.001	
Reciprocity	25.41	0.22	<0.001		8.6	0.23	<0.001	

## Discussion

In this study, we investigated confidant social networks within a cohort of 660 community-dwelling older adults and found a marginally significant depressive symptom homophily. When we examined specific dimensions of depression, we detected significant apathy homophily but not suicidal ideation homophily.

While many studies have addressed the contribution of social networks to the well-being and longevity of older adults, little research has been done to elucidate depressive symptom homophily in older adults' social networks. Our findings of depressive symptom homophily in the confidant social network of older adults is consistent with those of studies that highlighted similarity as a strong foundation in friendship and marriage (11). One of the more popular explanations for homophily is that discovering individuals who are similar to one's characteristics leads to perceived consensus support for who they are, and feeling comfortable in establishing and maintaining the relationship (35). A study on adolescents reported that depressed individuals were not rejected by peers but, over some time, they withdraw from peers who were not depressed (12). A study of undergraduate students investigated the time spent on interactions with friends and found that depressed individuals spend less time with friends but longer time with individuals who display a similar level of depressive symptoms (10). As apathy is one of the most common neuropsychiatric symptoms in later life (36), we hypothesized that apathy homophily might be a result of apathetic older adults withdrawing themselves from other more energetic older adults and spending longer a time with similarly apathetic older adults. Another possible explanation for the homophily phenomenon in social networks is social influence. Christakis et al. have demonstrated in a Framingham Heart Study that families, friends, and neighbors who spend time together become more similar to each other over time in terms of both positive and negative emotions (13, 14, 37).

On the other hand, in this study, we did not observe suicide ideation homophily. Pachucki et al. found that depressed young teenagers were more likely to withdraw from the mainstream group in their classes and that depressive symptoms did not spread over time among connected individuals (38). The authors hypothesized that social influence was weak in this age group because they were not able to pick up signs and symptoms of depression. Our participants might not be able to pick up suicide ideation in their community members. Suicide stigma is highly prevalent in Japan, more so than in other countries, such as Australia (39). Japanese often consider suicide as a way to avoid shame or dishonor, and those with suicide ideation fear lifelong discrimination or ostracization (40). People with such fear are reluctant to seek help, particularly those in rural communities where anonymity is lacking (41).

We must note the limitations of our study. First, we limited our target to the community-dwelling older adults under the national insurance system, and not all of them participated in the study. Although a multi-national study reported the number of confidants among older adults was generally small, less than three on average even when including non-older adults, such as children (42), our sampling strategy and consequence may have contributed to a low network density (i.e., 0.001). Future research that receives research cooperation from most of the community members and measures confidant networks of various ages could further verify our findings. Second, our study was cross-sectional. Hence, we cannot differentiate the direction, that is, whether depressive symptoms homophily induced confidant social network, or the other way around. A further longitudinal study is needed to elucidate the directionality of this association. Last but not least, the likelihood of a tie we reported in this study is based on ERGM with MCMC simulation in one small city in Japan. Although the number of scientific literature employing ERGM is rapidly increasing over the last 10–15 years (43), and ERGM is one of the most popular modeling in social network analysis (32), we cannot deny the possibility that the estimated value would differ if we employ a different modeling. Also, our participants were similar with respect to mean age and sex proportions, but they were coming from relatively urbanized areas than rural areas of a community compared with the targeted population. Different populations from different communities, such as more rural or more urban, may have different social networks. Further studies are needed to confirm if our findings apply to other settings.

Despite the limitations, our findings suggest that older adults may have difficulty in receiving help to alleviate their apathetic depressive symptoms from close social ties of their age. Geriatric clinicians, welfare specialists, and volunteers may express empathy to apathy dimension of depression to build and maintain a confidant relationship and cooperate to ensure older adults receive needed support and companionship over time (44, 45). Furthermore, they may invite or connect with older adults to participate in group-based interventions with their closest social ties to increase emotional viability.

In conclusion, Japanese community-dwelling older adults are likely to form confidant social networks with other older adults who have a similar level of apathetic depressive symptoms. Depressive symptom homophily in the social networks of older adults in the community highlights the importance of targeting not only older adults but also their confidants in promoting good mental health in later life.

## Data availability statement

The study materials, analytic methods, and data are available from the corresponding author on reasonable request.

## Ethics statement

The studies involving human participants were reviewed and approved by the research Ethics Committee at Tokyo Medical Dental University. The patients/participants provided their written informed consent to participate in this study.

## Author contributions

AM and TF conceived study and analyzed and drafted. AM collected data. AM, YT, KT, and TF finalized the manuscript. All authors were involved in writing the paper and had final approval of the submitted and published versions.

## Funding

This study was supported by Health, Labor, and Welfare Policy Research Grants (H29-Seisaku-Shitei-004), Grants-in-Aid for Scientific Research from the Japan Society for the Promotion of Science (JSPS KAKENHI 16H06768, 17K19794, and 19K10639), and Tokyo Medical and Dental University (2017).

## Conflict of interest

The authors declare that the research was conducted in the absence of any commercial or financial relationships that could be construed as a potential conflict of interest.

## Publisher's note

All claims expressed in this article are solely those of the authors and do not necessarily represent those of their affiliated organizations, or those of the publisher, the editors and the reviewers. Any product that may be evaluated in this article, or claim that may be made by its manufacturer, is not guaranteed or endorsed by the publisher.
